# Unveiling metabolic dysfunction-associated fatty liver disease: Knowledge gaps and attitudes among Lebanese university students

**DOI:** 10.1371/journal.pone.0306825

**Published:** 2024-08-02

**Authors:** Mohamad Abdelkhalik, Samah Al Tawil, Adam El Fouani, Nour Allakiss, Lama Mattar, Wissam H. Faour, Rajaa Chatila

**Affiliations:** 1 Gilbert and Rose-Marie Chagoury School of Medicine, Lebanese American University, Byblos, Lebanon; 2 Lebanese American University Medical Center – Rizk Hospital, Beirut, Lebanon; 3 Natural Sciences Department, School of Arts and Sciences, Lebanese American University, Byblos, Lebanon; University of Montenegro-Faculty of Medicine, MONTENEGRO

## Abstract

**Background:**

Metabolic dysfunction-associated fatty liver disease (MAFLD) is a rapidly growing global health problem. Despite its growing incidence and potential for significant repercussions, MAFLD is still widely misunderstood and underdiagnosed.

**Aim:**

The purpose of this study was to investigate MAFLD-related knowledge, attitudes, and risk profiles among university students aged 17 to 26.

**Methods:**

A cross-sectional study with 406 university students in Lebanon, equally distributed among males and females, was conducted using a questionnaire that includes demographics, medical information, dietary habits, physical activity, and MAFLD-related knowledge and attitudes.

**Results:**

The findings demonstrated a significant lack of knowledge regarding MAFLD, with more than half of participants (54.7%) having no prior knowledge of the illness. Students exhibited unhealthy lifestyle behaviors such as smoking (68%), insufficient physical exercise (44.1%), and poor food habits (52.5%). Having a family history of heart disease, personal history of diabetes mellitus, a balanced diet and prior knowledge of the disease were associated with a higher knowledge score (p<0.05). A higher attitude score existed among those who have a personal or family history of chronic diseases and those who have a prior negative impression about the disease, prior knowledge of the disease, and those who are physically active (p<0.05).

**Conclusion:**

Despite knowledge gaps, university students in Lebanon have, in general, an appropriate and positive attitude towards MAFLD. We recommend the introduction of focused educational interventions to address the necessity of lifestyle changes among university students and the community as a whole. Developing comprehensive MAFLD prevention methods requires future studies in different age groups and demographics

## Introduction

Metabolic dysfunction-associated fatty liver disease (MAFLD) refers to a group of liver diseases that range histologically from simple steatosis (fatty liver) to non-alcoholic steatohepatitis (NASH), which is characterized by inflammation and liver cell destruction. MAFLD is often linked with obesity, insulin resistance, metabolic syndrome, and sedentary lifestyle. Although MAFLD was previously believed as a relatively benign illness, new research has highlighted that MAFLD may progress to severe liver disease, including liver cirrhosis and hepatocellular carcinoma [1]. MAFLD also contributes to health problems in organs other than the liver. MAFLD is specifically associated with the development of cardiovascular diseases, type 2 diabetes mellitus (T2DM), and chronic kidney disease [2–4].

MAFLD prevalence has reached epidemic proportions worldwide, and is currently regarded as the most emerging liver disease in terms of both mortality and morbidity [5]. A thorough meta-analysis found that MAFLD affects around 25% of the world’s population, making it one of the most prevalent liver illnesses globally [6]. The disease is especially widespread in Western nations, where high-calorie meals and sedentary lifestyles are increasingly common. For instance, it has become the most prevalent chronic liver disease in the United States, affecting an estimated 30% of the population [7].

Despite its astounding frequency and its potential for serious health implications, MAFLD remains underdiagnosed and misunderstood in the general population [8, 9]. A substantial portion of this problem may be attributable to the asymptomatic character of the disease in its early stages. Individuals with MAFLD often have no symptoms until the illness has progressed to more severe stages. This absence of symptoms, along with a lack of awareness, leads to a false sense of well-being and a reluctance to seek medical assistance [8].

Furthermore, there is a widespread lack of understanding of the risk factors for MAFLD. Many people are unaware that obesity, T2DM, and metabolic syndrome all enhance their vulnerability to the illness [10]. Moreover, certain risk factors are not as visible as obesity or T2DM. In one research conducted in rural India, lean MAFLD patients accounted for more than half of all MAFLD cases [11]. This demonstrates the disease’s intricacy as well as the multitude of physical and environmental risk factors that may contribute to its development.

MAFLD is becoming a rising health problem across the Middle East, and the condition appears to disproportionately affect countries in the region, Lebanon included. According to Younossi et al. (2016), the Middle East has the highest incidence of MAFLD (32%) globally [6]. Further, the prevalence of MAFLD is expected to rise from 25.7% to 31.7% in Saudi Arabia by 2030, and from 25% to 30.2% in the United Arab Emirates [12]. Additionally, several studies in Iran showed that the prevalence of MAFLD ranges from 3 to 44% [13–16]. In Lebanon, despite the expected high prevalence, knowledge about MAFLD and its prevalence remain understudied. One study, which sought to analyze the dietary patterns that may predict MAFLD in the Lebanese population, discovered that a diet high in fruits, meat and fast food are the main potential risk factors for MAFLD [17].

When addressing the issue of raising awareness and comprehension of MAFLD, the youth emerge as a pivotal demographic. However, despite the critical role of university students, research on the subject has mainly focused on graduate students in the healthcare field [18, 19]. Given the misunderstanding and lack of awareness of MAFLD as well as its increasing prevalence, awareness of undergraduate students from all fields should be assessed as well. When it comes to MAFLD associated mortality and morbidity, prevention is a key factor. Patients with fibrotic MAFLD have a threefold risk increase in all-cause mortality compared to patients with non-fibrotic MAFLD [20]. Hence, understanding the burden and awareness of MAFLD in young individuals might allow for earlier intervention, preventing the development of advanced liver disease and cirrhosis.

Young adults, including university students, are an underserved group with a clinical blind spot when it comes to identifying MAFLD, with only a few studies existing in the United States, Australia, and the United Kingdom [21–24]. They often have asymptomatic illness and, as a group, see doctors less frequently [25]. Interestingly, an Australian study estimated MAFLD prevalence to be around 15.2% in adolescents aged between 17-18 [21]. Another study from the United States showed the prevalence of MAFLD, among American young adults, has increased from 9.6% to 24% in the period of between 1988 till 2010 [24]. This sheds the light on the importance of targeting this age group when it comes to MAFLD knowledge.

The evident scarcity of research on the knowledge and attitudes towards MAFLD in Lebanon, in a setting of worldwide and locoregional dramatic rise of the disease’s health and socioeconomic burden, makes gaining insight about this condition, especially by young adults, a critical objective [19]. In addition, by having contextually relevant knowledge about the risk factors and behavior towards the disease among university students, public health professionals and physicians will be better equipped to address this rapidly emerging and grave condition. Therefore, this study aimed to assess the knowledge of Lebanese university students about MAFLD, evaluate their risk profile for MAFLD by studying their dietary habits and physical activity level, and examine the attitudes of these students towards the disease.

## Methods

### Study design

This is a cross-sectional study that was conducted between December 2023 and January 2024, following the approval of the Lebanese American University (LAU) Institutional Review Board. All participants provided written consent before they could participate. Participation was voluntary and participants could opt out at any time if they felt uncomfortable. All collected data had no personal identifiers and are kept confidential and are accessible only to the research group.

### Target population

The target population comprised university students aged 17-26 years enrolled in various Lebanese universities across Lebanon. Exclusion criteria included populations outside the specified age range.

Given the absence of similar studies on MAFLD knowledge in Lebanon, we utilized the CDC Epi-info seven software to calculate the minimum sample size. With an expected frequency of 50%, a minimum of 384 participants was determined necessary to achieve a 95% confidence interval, with a 5% margin of error and 80% power.

### Data collection

Convenient sampling was employed among university students from diverse institutions across Lebanon. Sampling was conducted through online platforms including Instagram, Facebook, Twitter, LinkedIn, and WhatsApp, as well as the snowballing method. Participants who provided informed consent were included in the study. The data was collected via a self-administered online questionnaire, available in both Arabic and English and estimated to take 10-15 minutes to complete.

### Study instrument

The study instrument was a validated questionnaire adapted from a study done in China (10) (Chen et al., 2019). The questionnaire was divided into five sections: demographics, medical information including Body Mass Index (BMI), socioeconomic status, and familial history of diseases associated with MAFLD.

To assess disease knowledge, a scale comprising twenty-seven questions, adapted from (10), was employed. Our team supplemented this scale with two additional questions on risk factors and three questions regarding disease progression, lifespan comparison, and the impact of early diagnosis and treatment. Questions on various aspects of MAFLD were awarded one point when answered correctly. These included prevalence (n=1), reversibility (n=1), health consequences (n=4), symptoms (n=5), diagnosis (n=3), treatment (n=7), and risk factors (n=9). Questions were in true-false and MCQ format. Physical activity was assessed through a single-item inquiry.

The assessment of participants’ dietary habits was structured into two components. The first component encompassed the adoption of a validated tool, the "Starting the Conversation: Diet "Scale. This scale organized response options into three columns: the left column represented the most healthful dietary practices (score 0) and the right column represented the least healthy (score 2). The center column presented dietary practices that were less healthy (score 1). Responses of the participants to individual questions in the survey were summed up and the final score, which was a number between 0 and 16, was obtained by adding all the item scores. Lower scores indicated a healthier diet, while higher scores revealed the areas that had the greatest room for improvement.

Furthermore, our assessment included extra questions to explore the dietary behaviors that were associated with MAFLD risk factors and management modalities. This provided a better insight into the dietary patterns of the participants and their relevance to MAFLD susceptibility and management. To assess the attitudes regarding MAFLD, a 17-question scale was developed by our team to assess participants’ attitudes regarding MAFLD. The questions were in Likert scale format “1-5” and the total score was conducted by summing up responses to individual questions, giving a final score between 7 and 85. The survey was initially developed in English, and content was checked for its accuracy by all authors. Pilot testing was conducted on 20 volunteers to ensure the content validity of the assessment tool.

### Statistical analysis

Descriptive statistics were calculated using mean and standard deviation (SD) for normally distributed continuous variables, median and interquartile range for continuous variables lacking a normal distribution and counts and percentages for categorical variables. Continuous data were tested for normality using the Shapiro–Wilk test. Comparison between groups was made using the unpaired t-test and Analysis of Variance (ANOVA) test if variables were normally distributed and nonparametric methods (Mann–Whitney U test or Kruskal-Wallis test) if variables were not normally distributed. As for categorical variables, the Pearson chi-square test was used, and when the expected values within cells were <5, the Fisher exact test was the substitute. Multivariable logistic regression analysis was performed to identify predictors of both MAFLD knowledge, and attitudes scores. Variables that were significant on univariate analysis (p<0.05) were included in the multivariate analysis. In all cases, a p-value ≤0.05 was considered significant.

### Ethical considerations

Patients’ records were treated with confidentiality. No personal information was disclosed. Anonymity was preserved in a way that no names were revealed throughout the study and any information that would possibly unravel the patient’s identity was removed. This study was approved by the Institutional Review Board (IRB) of the Lebanese American University (LAU) and registered under the ***IRB #*:** *LAU*.*SOM*.*RC1*.*19/Dec/2023*, including the mentioned aged in the study and students happened to be 17-year old students were approached to fill a consent.

## Results

### Socio-demographic characteristics

A total of 406 participants participated in this study. Males and females were equally distributed (50.7 vs. 49.3%). The mean age was 22.3±2.32 and the mean BMI was 26.06±4.44 with 67.3% being overweight or obese. The majority were single (91.1%), with middle economic/social status and not specialized in the healthcare field (62.6%). The majority of participants were smokers, with only 32% being non-smokers. Almost one third of them had one of the metabolic diseases, T2DM, dyslipidemia, fatty liver disease or hypertension with an even higher proportion of these diseases reported in their families exceeding 50% for hypertension (60.3%), T2DM (56%) and dyslipidemia (594%). More details on the socio-demographic and medical data are found in Table 1.

**Table 1:** Sociodemographic characteristics of study participants.

Characteristics	N (320)	Percentage (%)
Residence Governorate		
Beirut	110	27.1
Mount-Lebanon	85	20.9
North	67	16.5
South	62	15.3
Bekaa	82	20.2
**Age (years)**		
18-21	182	44.8
22-25	181	44.6
26-29	43	10.6
**BMI**		
<24	133	32.8
24-28	146	36
>28	127	31.3
**Gender**		
Males	206	50.7
Females	200	49.3
**Are you specialized in healthcare?**		
No	254	62.6
Yes	152	37.4
**Nationality**		
Lebanese	395	97.3
Other	11	2.7
**Social status**		
Single	370	91.1
Married	32	7.9
Divorced/ Separated	4	1
**Economic Status**		
Low	41	10.1
Middle	206	50.7
High	159	39.2
**Smoking Status**		
I have never smoked	131	32.3
Occasional Smoker	29	7.1
Current cigarette/e-cigarette smoker	186	45.8
Current nargileh smoker	49	12.1
**Number of cigarettes or e-cigarettes/day**		
Less than 10	37	9.1
10-20	79	19.5
More than 20	112	27.6
**Number of waterpipes/day**		
Less than 1	24	5.9
1-2	50	12.3
More than 2	115	28.3
**Medical History**		
Diabetes Mellitus	142	35
Hypertension	133	32.8
Hyperlipidemia	140	34.5
Heart Disease	126	31
MAFLD	105	25.9
**Family History**		
Diabetes Mellitus	232	57.1
Hypertension	245	60.3
Hyperlipidemia	241	59.4
Heart Disease	179	44.1
MAFLD	112	27.6
**Knowledge of MAFLD prior to survey**		
No	222	54.7
Yes	184	45.3
**Source of information about MAFLD**		
My primary physician	77	19
My family/friends	90	22.2
Books, magazines, newspapers	58	14.3
Internet	230	56.7
Television, radio or public advertisements	35	8.6
Health Education in community or hospital settings	34	8.4
Others	81	20
**Negative Impression of MAFLD prior to survey**		
No	289	71.2
Yes	110	27.1
	**Mean**	**SD**
**Age**	22.3	2.32
**BMI**	26.06	4.44

*Note*. Metabolic Dysfunction-Associated fatty liver disease (MAFLD), Body Mass Index (BMI)

### Dietary habits and physical activity

Responses to questions on dietary habits and physical activity are shown in Table 2. More than half of the participants (52.5%) perceived their diet to be unhealthy. Most participants (42.9%) ate more than three meals a day on average, and a similar percentage reported snacking often between meals. 37.9% of the participants reported having more than 5 sugary drinks per week. Of the participants, 44.1% perceived themselves as being physically inactive and less than a quarter of the participants exercised more than 4 times a week. Analysis of responses to Starting the Conversation (STC) diet score revealed a mean score of 9.19±5.3.

**Table 2:** Responses to survey questions on diet and physical activity.

Characteristics	N (320)	Percentage (%)
**Self-perception of Diet**		
Unhealthy	213	52.5
Healthy	71	17.5
Sometimes healthy	71	17.5
Unsure	51	12.6
**Average meal (s) per day**		
<3	78	19.2
3	154	37.9
>3	174	42.9
**Average number of desserts per day**		
Never	30	7.4
Rarely	134	33
Occasionally	75	18.5
Often	167	41.1
**Average number of sugary beverages per week**		
0	36	8.9
01-Feb	133	32.8
03-May	83	20.4
> 5	154	37.9
**Snacking between meals**		
Never	25	6.2
Rarely	121	29.8
Occasionally	88	21.7
Often	172	42.4
**Physical activity**		
Not active	179	44.1
Once or twice per week	125	30.8
3-4 times per week	70	17.2
More than 4 times per week	32	7.9
**STC score (Mean ± SD)**	9.19±5.3	

### MAFLD-related knowledge and attitude scores

The mean scores of the knowledge and attitude were 16.43 ± 3.32, and 42.37 ± 11.27, respectively. Overall, most of the respondents (54.7%) did not know about MAFLD before this survey and only 27.1% had a negative impression about the disease prior to the survey. Among those who knew about the disease, 56.7% got their information from internet websites.

**Fig 1:**
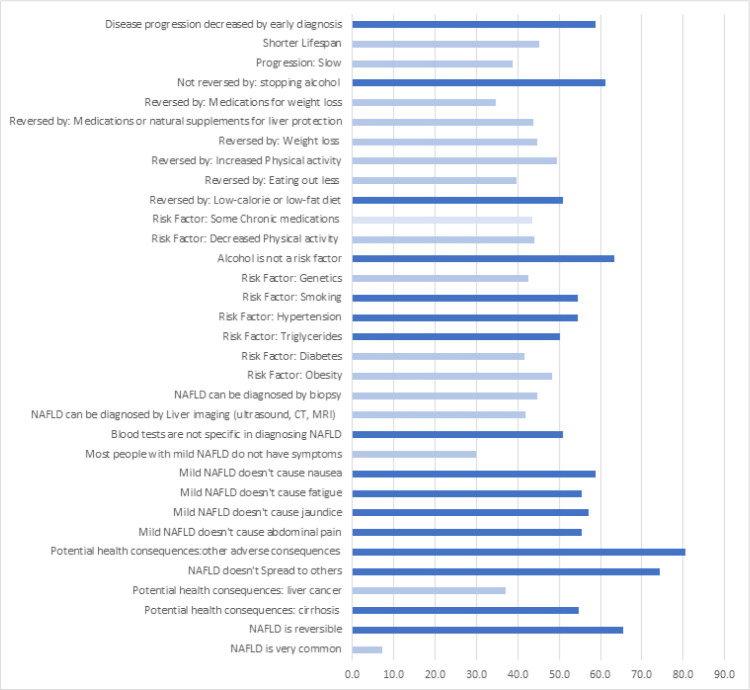
Percentage of participants who responded correctly to survey questions on MAFLD knowledge. Lighter-colored bars indicate specific questions in which less than 50% of participants answered correctly.

The percentage of participants who responded correctly to each MAFLD knowledge question is shown in Fig 1. 37% of study participants were aware that MAFLD can cause cancer. More than 50% of the participants knew that MAFLD is reversible, can cause cirrhosis, and is not treated by stopping alcohol. The majority of the participants (90%) knew that MAFLD does not spread to others, but only 40 to 45% are aware that it can be diagnosed by imaging or liver biopsy. Although almost half of them knew obesity is a risk factor for MAFLD, only 41% recognized T2DM. Half of the participants knew treatment options for MAFLD include a reduced-calorie diet and recognized increased physical activity is an option. Only 30% of the participants recognized most patients with MAFLD are asymptomatic. The question with the fewest correct responses pertained to prevalence of MAFLD, with only 7% of participants answering this question correctly.

**Fig 2:**
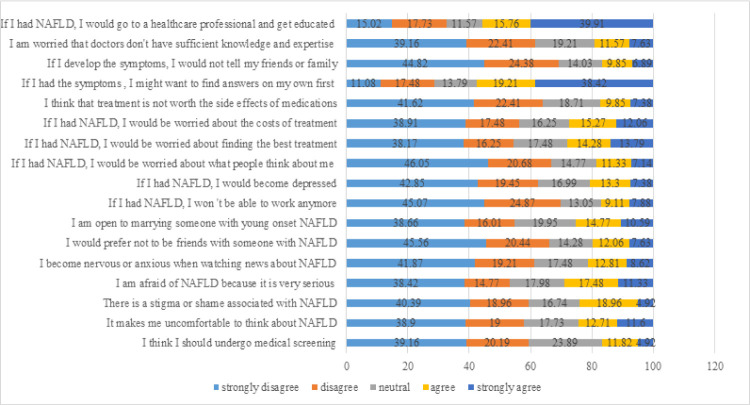
Percentage of participants with different attitudes regarding MAFLD.

When asked about their attitudes, the majority of the participants (38.91%) strongly disagreed that there is stigma or shame associated with MAFLD. Less than one third of people felt uncomfortable to think about MAFLD (23.88%) and became nervous while watching news about the disease (21.43%). Only 28.81% of the participants agreed or strongly agreed that they were afraid of MAFLD because it is a serious disease and 46.05% were worried about what people might think of them if they get the disease. While 66% of the participants agreed that they would become friends with someone who has MAFLD, only 38.66% were open to marrying someone with MAFLD. Moreover, 39.91% agreed to go to a healthcare professional and get educated on the disease and 38.82% would search for more information about the disease on their own. More details of the attitude score are found in Fig 2.

### Difference of knowledge and attitudes scores according to participants’ characteristics

#### Social and medical determinants of knowledge and attitudes scores: Bivariate analysis

Table 3 shows variables associated with high MAFLD knowledge scores. On bivariate analysis, being a female, living in Beirut, having a prior knowledge about MAFLD and having a negative impression about it were significantly associated with higher knowledge score. Moreover, non-smokers or occasional smokers along with those who had a background about the disease from a healthcare setting scored higher knowledge scores than others. As for the attitude score, a higher score (ie. worse or negative outlook) was seen in females, married, non-healthcare professionals, low economic status and those residing in Beirut. Similarly, a higher attitude score was also seen among those who had a personal or family history of chronic diseases especially MAFLD and among those who have a prior negative impression about MAFLD. To add, being physically active and healthy were significant predictors of higher MAFLD knowledge and attitude scores, while the number of sugary beverages, desserts and snacks per week had a negative association with both knowledge and attitude (p<0.001). Furthermore, a moderate negative correlation was seen between STC score and each of knowledge and attitudes score while a higher knowledge score was positively associated with attitudes score.

**Table 3:** Difference of mean scores of the different scales depending on socio-demographic and profession characteristics.

Variable	Knowledge scale (Mean ± SD)	*p-value*	Attitude scale	*p-value*
**Gender**				
Male	15.31±3.34	**0.001**	40.63±11.07	**0.001**
Female	16.9±3.25		44.18±11.22	
**Marital status**				
Single	16.16±4.44	0.508	41.76±11.22	**0.002**
Married	15.30±3.34		48.84±10	
Divorced/separated	16.35±3.50		47.75±9.74	
**Healthcare professional**				
No	16.38±3.13	0.118	44.74±11.11	**<0.001**
Yes	15.64±3.63		38.43±10.44	
**Nationality**				
Lebanese	16.00±3.31	0.06	42.35±11.3	0.79
Others	19.64±2.54		43.27±10.47	
**Economic status**				
Low	16.09±2.87	0.71	45.51±12.64	**<0.001**
Middle	16.27±3.53		39.41±10.55	
High	15.88±3.17		45.42±10.84	
**Residence**				
Beirut	18.57±3.47	**<0.001**	45.15±9.92	**0.001**
Others	15.19±3.07		41.35±11.58	
**Did you know about the disease prior to this survey?**	** **	** **	** **	** **
No	15.28±3.07	**<0.001**	41.63±11.68	0.139
Yes	18.25±3.5		43.29±10.72	
**Prior impression prior to survey**				
Negative	18.4±3.16	**0.006**	46.6±9.01	**<0.001**
Positive	17.05±3.17		40.75±11.64	
**Occasional smoker**				
No	15.9±3.3	**<0.001**	42.25±11.18	0.422
Yes	18.85±3.25		44±12.51	
**Former smoker**				
No	16.1±3.22	0.438	42.56±11.22	0.273
Yes	15.4±4.45		40.28±11.87	
**Current cigarette smoker**				
No	17.55±3.65	**<0.001**	43.05±10.99	0.19
Yes	14.36±2.42		41.58±11.58	
**Number of cigarettes per day**				
<10	17.08±3.20	**<0.001**	46.65±8.88	**<0.001**
10-20	15.39±3.97		45.58±11.14	
>20	12.90±2.54		36.14±10.28	
**Number of arguileh per day**				
<1	16.87±2.75	**<0.001**	44.5±9.97	**<0.001**
1-2	15.98±3.07		43.02±11.04	
>2	14.41±2.14		36.76±10.82	
**Prior disease knowledge source: Primary physician**	** **	** **	** **	** **
No	16.05±3.43	0.64	40.97±11.49	**<0.001**
Yes	16.32±2.84		48.42±7.88	
**Prior disease knowledge source: Family/friends**	** **	** **	** **	** **
No	15.94±4.50	0.113	41.32±11.32	**<0.001**
Yes	16.68±3.59		46.09±10.33	
**Prior disease knowledge source: Books, magazines, newspapers**	** **	** **	** **	** **
No	16.02±4.37	0.36	41.9±11.41	**0.037**
Yes	16.59±4.28		45.24±10.01	
**Prior disease knowledge source: Internet**	** **	** **	** **	** **
No	16.77±4.54	**0.008**	42.92±11.33	0.398
Yes	15.61±4.12		41.97±11.23	
**Prior disease knowledge source: TV/radio**	** **	** **	** **	** **
No	16.12±3.33	0.377	41.96±11.33	**0.014**
Yes	15.88±3.4		46.86±9.64	
**Prior disease knowledge source: Health education in the hospital/community**	** **	** **	** **	** **
No	15.91±4.31	**0.003**	42.17±11.31	0.221
Yes	18.24±4.35		44.65±10.73	
**Previous history - Diabetes**				
Yes	17.38±3.42	**<0.001**	44.77±10.58	**<0.001**
No/I don’t know	13.72±2.45		37.94±11.22	
**Previous history - HTN**				
Yes	17.32±4.29	**<0.001**	44.5±10.53	**<0.001**
No/I don’t know	13.64±3.35		38.03±11.54	
**Previous history - Hyperlipidemia**				
Yes	17.26±4.26	**<0.001**	44.6±10.46	**<0.001**
No/I don’t know	13.90±3.64		38.16±11.59	
**Previous history – heart disease**				
Yes	17.31±4.29	**<0.001**	44.5±10.55	**<0.001**
No/I don’t know	15.44±3.17		37.67±11.43	
**Previous history – Liver disease**				
Yes	16.98±3.37	**<0.001**	44.44±10.59	**<0.001**
No/I don’t know	13.61±2.61		36.49±11.15	
**Family history - Diabetes**				
Yes	17.18±4.15	**<0.001**	44.02±10.99	**0.012**
No/I don’t know	15.28±4.41		41.15±11.34	
**Family history - HTN**				
Yes	16.95±3.41	**<0.001**	43.71±10.93	0.053
No/I don’t know	15.55±3.23		41.5±11.43	
**Family history - Hyperlipidemia**				
Yes	16.76±4.24	**0.013**	44.82±10.33	**<0.001**
No/I don’t know	15.65±4.39		40.71±11.6	
**Family history – heart disease**				
Yes	17.11±3.44	**<0.001**	44.48±10.7	**<0.001**
No/I don’t know	15.57±2.97		39.72±11.45	
**Family history – Liver disease**				
Yes	17.03±4.27	**<0.001**	44.26±10.65	**<0.001**
No/I don’t know	13.74±3.61		37.44±11.42	
**Self-perception of diet**				
Not Healthy	15.02±3.79	**<0.001**	41.42±11.76	**<0.001**
Occasionally healthy	19.65±3.49		47.78±9.8	
Always Healthy	18.64±3.92		45.63±9.16	
**How many meals do you consume per day?**	** **	** **	** **	** **
≤3	18.03±3.63	**<0.001**	47.84±9.26	**<0.001**
>3	13.55±3.92		35.09±9.45	
**Average number of desserts per week**	** **	** **	** **	** **
Never	16.31±2.51	**<0.001**	51.13±10.35	**<0.001**
Rarely/Occasionally	17.81±3.70		47.19±9.42	
Often	13.98±4.44		34.77±8.87	
**Average number of sugary beverages per week**	** **	** **	** **	** **
≤3	18.15±3.03	**<0.001**	47.51±9.48	**<0.001**
>3	14.63±3.22		38.72±11.03	
**Intake of snacks**				
Never	16.28±2.91	**<0.001**	49.44±11.71	**<0.001**
Occasionally	17.92±3.57		47.35±9.56	
Often	13.96±4.36		35.3±9.07	
**Physical activity**				
Not active	15.07±2.89	**<0.001**	36.58±10.32	**<0.001**
Somewhat active	17.34±3.19		47.26±9.72	
Very active	18.46±3.54		45.09±10.2	
**Correlations (r2)**				
** Age**	-0.11	**0.036**	0.06	0.814
** BMI**	-0.27	**<0.001**	-0.13	0.008
** STC score**	-0.6	**<0.001**	-0.54	**<0.001**
** Knowledge score**	-----	-----	0.31	**<0.001**

*Note*. Television (TV), Hypertension (HTN), Body Mass Index (BMI). Data are considered significant with a *p* < .05

#### Multivariable analysis

A linear regression was performed for multivariable analysis and knowledge and attitudes scores were used as the dependent variables. The Omnibus Tests of Model Coefficients was found significant (*p*<0.001) suggesting that the model is fit and suitable to the data and that at least one of the independent variables was considered as significant in explaining the dependent variable. The Hosmer and Lemeshow goodness-of-fit test emphasized that the model is fit with its data and that the observed event rates match the expected ones. The overall percentage from the classification table was 78.5% suggesting that the entered variables could explain more than 50% of the variability of the dependent variable. The Nagelkerke R square values revealed that 44.3% and 60.4% of the variation of knowledge score and attitude scores is due to the variation of the independent variables included in the model respectively.

The first linear regression taking the knowledge scale as the dependent variable showed that the increasing number of meals per day and an increasing STC score were associated with a lower knowledge score (ORs = -2.09 and -0.17 respectively, p<0.001). However, having a family history of heart disease, personal history of DM, being sometimes healthy and a prior knowledge of MAFLD especially using the internet as a primary source of information were associated with a higher knowledge score. (Table 4, model 1).

**Table 4:** Multiple linear regression of knowledge and attitudes scores.

	Unstandardized Beta	Standardized Beta	p-value	95% Confidence Interval	
**Model 1. Dependent variable: Knowledge score**	** **	** **	** **	** **	** **
Number of meals per day	-2.28	-0.71	**0.002**	-3.692	-0.885
Family History of Heart Disease	2.26	0.628	**<0.001**	1.024	3.506
Heaving a healthy diet (sometimes vs. never)	3.259	0.887	**<0.001**	1.506	5.011
Previous knowledge about MAFLD	1.043	0.455	**0.023**	0.144	1.941
Prior disease knowledge source: Internet	1.611	0.577	**0.006**	0.47	2.752
Previous History – Diabetes Mellitus	2.179	0.692	**0.006**	0.812	3.547
Total STC score	-0.17	0.066	**<0.001**	-0.3	-0.038
**Model 2. Dependent variable: Attitudes score**	** **	** **	** **	** **	** **
Number of desserts per week (occasional vs. never)	-8.379	-0.377	**0.002**	-13.72	-3.037
Total STC score	-0.34	-0.164	**0.046**	-0.673	-0.006
Total knowledge score	0.64	0.171	**0.003**	0.23	1.17
Previous knowledge about MAFLD (My primary physician)	5.409	0.158	**0.006**	1.571	9.246
Number of snacks per day (occasional vs. never)	-10.747	-0.456	0	-16.423	-5.071
Number of snacks per day (often vs. never)	-8.51	-0.378	**0.042**	-16.701	-0.318
Physical activity (very active vs. not active)	10.239	0.179	**0.001**	4.258	16.22
Physical activity (sometimes active vs. not active)	3.372	0.148	**0.026**	0.406	6.337

*Note*. Metabolic Dysfunction-Associated Fatty Liver Disease (MAFLD). Data are considered significant with a *p* < .05

The second linear regression taking the attitude scale as the dependent variable showed that prior knowledge of the disease, being physically active and a higher knowledge score were significantly associated with a higher attitude score, i.e. a more negative outlook towards MAFLD whereas an increasing intake of desserts and snacks and a higher STC score were associated with a lower attitude, i.e. a more positive or favorable outlook towards MAFLD (Table 4, model 2).

## Discussion

Despite the global MAFLD pandemic, with 30% of western population and anywhere between 25-44% in eastern ones suffering from the condition [26, 27], our study revealed that university students’ awareness about MAFLD and its harmful implications are lacking. More than 50% of participants had no prior knowledge of MAFLD and a mere 27% had a notion of the negative implications of the disease. However, as expected, those with personal or family history of metabolic or cardiovascular disease had more knowledge about MAFLD than those who did not. The degree of unawareness of MAFLD is surprising and of high concern, considering the population under study is young university students, an educated sector from across the 5 governorates of Lebanon, most of whom coming from middle (50.7%) to high income (39.25%) families. This population generally has higher awareness levels than the rest of the population, as highlighted by previous studies from the US and Korea that have shown that socioeconomic disadvantage is a risk for MAFLD [28–30].

### MAFLD risk factors

In addition to the lack of awareness, our results revealed a widespread issue of unhealthy lifestyle among students. Indeed, 52% of the students are aware of having unhealthy eating habits and more than 67.7% are current smokers of cigarettes or e-cigarettes (45%), nargileh (12.1%), or occasional smokers (7%). As emphasized in previous studies, smoking seems to be significantly associated with metabolic-associated fatty liver disease. This association was highlighted by data extracted from a meta-analysis containing 20 observational studies and showing that active and passive smoking increase the risk of MAFLD by 1.11 folds and 1.38 folds respectively [31]. In addition to smoking and dietary eating habits, physical activity is another modifiable risk factor not only for MAFLD but also for non-communicable diseases in general [32]. In this study, a high level of inadequate physical activity was reported, with 44.1% of the students being totally inactive and 30.8% exercising only once or twice per week. This falls short of the recommended level of physical activity advocated by the World Health Organization (WHO) of 150 minutes of moderate intensity activity or 75 minutes of high intensity activity per week complemented by twice a week strength training [33]. The level of sedentary lifestyle appears to be significantly higher than the level of physical inactivity worldwide of 31%, as reported by the WHO [34]. This finding was not particularly surprising, given that the transition to university life is characterized by longer sitting time during lectures and studying, with a reported sitting time of around 7.29 hours per day [35]. Other studies have indeed demonstrated that starting university is associated with weight gain, unhealthy eating, sleep problems, and lack of physical activity [36]. Such trends of inactivity are in direct opposition to what our students ought to be doing, knowing that a sitting time of 6-8 hours per day is considered a risk for all-cause and cardiovascular mortality [37, 38], and that the WHO has classified physical inactivity as the fourth leading risk factor for global mortality (WHO, 2010).

While our study does not address the prevalence of MAFLD per se, it does reveal a high prevalence of MAFLD risk factors among students, some of which being modifiable, including poor healthy lifestyles, smoking, unhealthy diet and limited physical activity. Similarly, to recent studies looking at the increasing rate of obesity among the youth, our results have demonstrated an alarming prevalence (67%) of participants who are overweight or obese. To add, around one third already reported already suffering from one or more of the metabolic or cardiovascular diseases, and 25% already suffer from MAFLD. Furthermore, more than 50% of participants had family history of cardiovascular and metabolic diseases including hypertension, dyslipidemia and T2DM which highlights both the role of genetics, as well as habits and sociocultural norms in the development of metabolic syndrome [39]. Bearing in mind that MAFLD is the hepatic manifestation of the metabolic syndrome, our data raises real concerns about the real prevalence of this silent disease among students, at a young age.

### Knowledge and attitude scores

Our findings revealed a variable degree of knowledge with regards to the risk factors, presentation, and treatment of MAFLD. In our study, only 27.1% of participants had a negative impression about MAFLD and only 30% of participants knew that the disease is mostly asymptomatic. Propensity to unhealthy lifestyles and behaviors may be explained in part by this clear lack of adequate understanding of the risks associated with the disease and its ambiguous and asymptomatic nature of the MAFLD [40]. While many variables including gender, place of residence, personal and family medical history, lifestyle, perception about diet and STC score were significantly associated with knowledge about disease, only having a healthy diet, a positive family history of heart disease, and prior awareness about MAFLD were positively associated with knowledge of MAFLD. This finding confirms that lack of knowledge, coupled with the aforementioned asymptomatic nature of the disease, are major barriers against changing health behaviors and having the adequate level of motivation to alter said behaviors [40].

When it comes to the attitudes of the students in our sample, both positive and negative ones were reported. They reported a willingness to seek professional help, a lack of intimidation about having the disease, not expecting to feel depressed in the advent of having the disease, and a willingness to be friends with people with MAFLD all of which are undoubtedly positive attitudes. However, 54.7% reported being unwilling to marry someone with MAFLD and only 16.74% felt that it was necessary for them to undergo screening for it. Here, the multiple regression done in our study highlighted the crucial role of having appropriate knowledge when it comes to shaping more positive attitudes. Similarly, a qualitative study conducted in the United States emphasized that the provision of informative patient educational materials can help address the ambiguity of the disease [40]. Moreover, a recent study conducted in the United Kingdom demonstrated that the treatment of mental health conditions, in addition to improving attitudes towards diet and physical activity, might positively affect attitudes towards MAFLD in the general population [41]

## Strengths and limitations

The first limitation we can mention is that, as is well known, relying on self-reported data is always prone to several types of biases, such as the recall bias and the social desirability bias, the latter being of particular relevance in responses related to physical activity, weight, and eating habits. Another possible limitation is that the study was conducted only on university students, all between 17 and 26 years old, which reduces the generalizability of our results. Nevertheless, this might also constitute a positive element of our study, since it targets a crucial sector of the society that is future leaders, decision makers and parents of tomorrow [32].

Being first of its kind to explore MAFLD awareness, risk factors, knowledge, and attitudes in Lebanon, we had to resort to a cross-sectional design, which inherently limits our ability to establish causal or temporal relationships among variables. Still, our study sets the grounds for future studies that aim towards a better understanding of disease progression, by relying on other research methodologies, such as longitudinal cohort designs.

## Conclusion and final recommendations

Our findings shed light on the importance of knowledge and awareness about MAFLD as an important motivator for adequate behavior and attitudes. It also reveals a visible need for a concerted effort at the level of universities, community, and government to tackle this public health threat. Indeed, the implementation of strategies that would promote increased knowledge about the disease, but also that tackle potential barriers against change in attitudes and existing social behaviors can go a long way in encouraging the adoption of a healthier lifestyle and better attitudes, as our study seems to indicate. For instance, at the level of universities, an important barrier to address would the obstacles against routine exercise faced by students, which may include, but is not restricted, to time constraints due to study load.

Based on the results of our study, here are a few recommendations we believe would have a positive impact on the state of MAFLD in Lebanon:

1. Universities need to put emphasis on sports as part of their curricula and tackle barriers that prevent adequate levels of physical activity, since, as it is now widely known physical activity is a crucial variable when it comes to preventing non-communicable diseases, including MAFLD.
2. At the level of the community, schools and universities, targeted educational programs on non-communicable diseases in general would be beneficial, but a focus on MAFLD and the metabolic syndrome in particular is necessary, given the rise in prevalence of these conditions and their increasingly heavy health and economic burden. These programs would emphasize the modifiable nature of the risk factors, and the possibility of prevention via a healthy lifestyle, proper eating habits, smoking cessation and physical activity. A variety of means may be used to relay this information, such as producing educational videos, conducting awareness campaigns, and sharing learning materials on social media platforms.
3. Lastly, this study calls for future longitudinal studies to explore the effect of intervention campaigns and increasing awareness of the disease, on the behaviors and attitudes of individuals with regards to MAFLD.
